# Quantifying the Role of Importation on Sustained Malaria Transmission in a Low-to-Moderate Burden Region of Southwest Uganda

**DOI:** 10.1093/infdis/jiag008

**Published:** 2026-01-06

**Authors:** Adrienne Epstein, Okiria Aramanzan, Isaiah Nabende, Tonny Max Kayondo, Michael Obbo, Robert Tumwesigye, Innocent Wiringilimaana, Monica Mbabazi, Brian A Kagurusi, Stephen Tukwasibwe, Isaac Ssewanyana, Isobel Routledge, Jessica Briggs, Amy Wesolowski, Bryan Greenhouse, Grant Dorsey, Emmanuel Arinaitwe, Isabel Rodriguez-Barraquer

**Affiliations:** Department of Medicine, University of California San Francisco, San Francisco, California, USA; Infectious Diseases Research Collaboration, Kampala, Uganda; Infectious Diseases Research Collaboration, Kampala, Uganda; Infectious Diseases Research Collaboration, Kampala, Uganda; Infectious Diseases Research Collaboration, Kampala, Uganda; Infectious Diseases Research Collaboration, Kampala, Uganda; Infectious Diseases Research Collaboration, Kampala, Uganda; Department of Medicine, University of California San Francisco, San Francisco, California, USA; Infectious Diseases Research Collaboration, Kampala, Uganda; Infectious Diseases Research Collaboration, Kampala, Uganda; Infectious Diseases Research Collaboration, Kampala, Uganda; Department of Medicine, University of California San Francisco, San Francisco, California, USA; Department of Medicine, University of California San Francisco, San Francisco, California, USA; Department of Epidemiology, Johns Hopkins School of Public Health, Baltimore, Maryland, USA; Department of Medicine, University of California San Francisco, San Francisco, California, USA; Department of Medicine, University of California San Francisco, San Francisco, California, USA; Infectious Diseases Research Collaboration, Kampala, Uganda; Department of Medicine, University of California San Francisco, San Francisco, California, USA; Chan Zuckerberg Biohub, San Francisco, California, USA

**Keywords:** malaria, importation, *Plasmodium falciparum*, Uganda, elimination

## Abstract

**Background:**

Parasite importation remains a challenge to malaria elimination. The extent to which human travel contributes to sustained transmission in low-to-moderate burden areas is poorly understood.

**Methods:**

We conducted a 14-month longitudinal cohort study in Southwest Uganda. A total of 1918 individuals from 400 households were followed bimonthly. Travel histories and household characteristics were collected through surveys. Symptomatic and asymptomatic *Plasmodium falciparum* infections were captured through health facility surveillance and household reports (for symptomatic cases) and qPCR (for asymptomatic infections). Multilevel logistic regression models estimated associations between travel and infection. Population attributable fractions quantified travel's contribution to malaria.

**Results:**

Over the study, 283 infections (244 symptomatic episodes and 39 asymptomatic infections) were recorded. Associations between travel and malaria varied spatially and temporally, with positive associations in lower transmission villages (OR = 4.38, 95% CI 1.80–10.64) and during periods of low transmission. Associations were strongest for short-distance trips to nearby areas of higher incidence. Population attributable fraction analyses suggested travel accounted directly for 14% of malaria cases in low-transmission villages overall, rising to 30% during periods of low transmission.

**Conclusions:**

Overnight travel contributed to malaria burden, particularly in low-transmission villages and during low seasons, highlighting the need for strategies that address both local transmission and importation.

## Teaser

Using longitudinal cohort and surveillance data, this study found that parasite importation through human travel contributes substantially to malaria burden in Southwest Uganda, particularly in low-transmission villages and during periods of declining local transmission.

Over the past 2 decades, malaria incidence and mortality in Africa have declined by 36% and 29%, yet the disease remains a major cause of illness and death, with an estimated 246 million cases and 569 000 deaths in 2023 [1]. As malaria burden decreases and becomes spatially heterogeneous—due to vector control and improved case management—importation of parasites through human travel has emerged as an obstacle to control and elimination [2–6]. Though frequently cited, the rate of importation remains largely speculative due to limited studies conducted across Africa.

Uganda has substantial heterogeneity in malaria transmission due to geographic targeting of control measures and environmental variation. While national burden remains high, the Ministry of Health has set subnational elimination goals based on local receptivity and transmission [7]. For example, the Kigezi sub-region in the southwest demonstrated the lowest parasite prevalence nationally in the 2018–2019 Malaria Indicator Survey (<1% among children under 5) [8] and is targeted for malaria elimination in the Uganda National Malaria Strategic Plan [9]. Yet within Kigezi, malaria burden is uneven, and the role of importation in transmission is not understood.

This study quantified the potential impact of importation on malaria burden over a 14-month period in Kamwezi subcounty, Kigezi sub-region, Uganda. Using data from a longitudinal cohort of 400 households linked to passive surveillance at a public health facility, we assessed the association between overnight travel and incident infection and estimated the proportion of cases attributable to travel, considering malaria burden at both travel destinations and local sites at the time of travel.

## METHODS

### Setting

**Figure 1.**
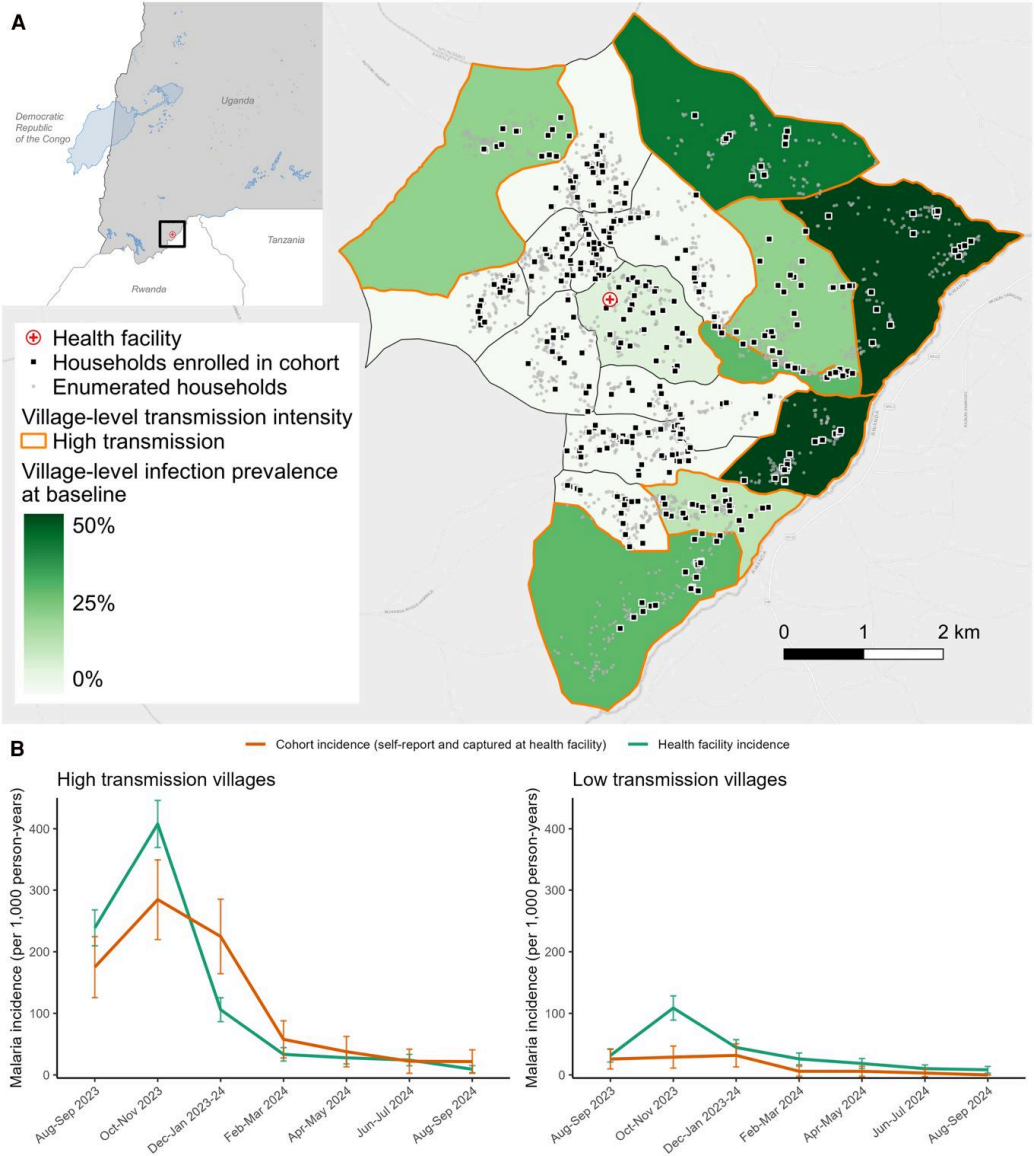
*A*, Map of households enrolled in cohort and village-level *P. falciparum* infection prevalence by qPCR. *B*, Malaria incidence over time measured through the cohort and at the health facility. Health facility-based incidence was calculated using cases from the study area that were captured at the facility as the numerator and a population estimate from an enumeration survey as the denominator. Incident cases in the cohort were captured at the health facility and via self-report. Map created using ArcGIS® basemaps licensed from Esri. © Esri. All rights reserved.

This cohort study was conducted in a 35 km^2^ area of Kamwezi subcounty, Rukiga District, Kigezi sub-region, Uganda, near the Rwanda border (Figure 1*A*). The area has low-to-moderate malaria transmission limited by altitude-related constraints. Epidemics have occurred, including during the first study year, but transmission has remained lower than in most of Uganda even before the scale-up of long-lasting insecticidal nets (LLINs) [10, 11]. Since 2013, the Ministry of Health has distributed LLINs every 3–4 years; the most recent (November 2023) used chlorfenapyr (chemical name, 4-bromo-2-(4-chlorophenyl)-1-(ethoxymethyl)-5-(trifluoromethyl)-1H-pyrrole-3-carbonitrile) + alpha-cypermethrin (chemical name, (S)-cyano(3-phenoxyphenyl)methyl (1R,3R)-3-(2,2-dichloroethenyl)-2,2-dimethylcyclopropane-1-carboxylate) nets. Indoor residual spraying has not been implemented in Rukiga District. Analyses cover 14 months (July 2023–September 2024) across 7 visits.

### Screening and Enrollment of Cohort Households

In January 2023, all households within a geographically defined area (n = 2904) were enumerated and mapped using GPS units to create a sampling frame (see Supplementary Figure 1 for profile). A household was defined as a permanent or semi-permanent dwelling serving as the primary residence for individuals or groups who cook and eat together. Village-level enrollment targets were proportional to household counts, and stratified random sampling by village was used to select households. Eligibility criteria included (1) at least 1 resident aged ≥18 years; (2) ≥80% of members willing to participate; (3) an adult providing informed consent; and (4) no plans to relocate within 2 years.

### Screening, Enrollment, and Follow-Up of Cohort Participants

All full-time residents (those intending to sleep primarily at the household for the next 6 months) were screened and enrolled if they were permanent residents and provided written informed consent (from parents/guardians for children aged 6 months-17 years, with assent for ages 12–17). The cohort was dynamic, allowing enrollment of new residents over time.

Households were visited every 2 months. During each visit, the head of household completed a questionnaire on household characteristics, malaria control interventions, and visitors in the preceding 2 months. Participants completed surveys on malaria illness, treatment, and travel (including destinations and nights away). A finger-prick blood sample (<1 mL, collected into microtainers and as dried blood spots) was obtained from participants at each visit. Analyses include 14 months (7 visits, July 2023–September 2024), excluding participants who permanently relocated or withdrew consent.

### Passive Health Facility-Based Surveillance

In parallel to the cohort, passive surveillance at the local health facility was conducted by enrolling patients with laboratory-confirmed *Plasmodium falciparum* (either through rapid diagnostic test or microscopy). For individuals enrolled in the cohort, identifiers were recorded, and a travel survey identical to that used in household visits was administered. This is the only health facility in the study area; at study enumeration, 85% of cohort participants reported care seeking at this facility. Monthly malaria incidence was estimated from health facility data, with the numerator defined as the number of laboratory-confirmed cases among residents of the study area and the denominator as the estimated population, calculated by multiplying the number of households recorded during the initial enumeration by the study's average household size at baseline (4.45).

### Laboratory Procedures

DNA was extracted from all household samples using the Tween-Chelex method [12]. Quantitative PCR (qPCR) was performed to detect *P. falciparum* and quantify parasitemia (parasites/µL) using a sensitive var gene acidic terminal sequence qPCR assay [13]. Each plate included a standard curve generated from cultured ring-stage parasites diluted in whole blood to 1–10 000 parasites/µL and spotted on filter paper in duplicate.

### Variable Definitions

#### Importation Exposure Variables

Given the nocturnal biting behavior of Anopheles vectors, we focused on overnight travel as the relevant exposure for *P. falciparum* importation. Recent overnight travel was defined as spending at least 1 night outside the village of residence in the prior 2 months. Travel-related exposures included (1) any overnight travel (binary); (2) travel to areas with higher district- or parish-level malaria incidence (binary, based on 2020 Malaria Atlas Project estimates [14]); and (3) travel duration in weeks (categorical; no travel, <1 week, ≥1 week). Among travelers to higher-incidence areas, we also evaluated (4) travel destination (district-level) and (5) travel distance (<25 km, 25–50 km, >50 km). Travel histories were obtained through health facility interviews for facility-detected cases and through bimonthly household surveys for others.

#### Outcome

The outcome for all analyses was individual-level incident symptomatic or asymptomatic *P. falciparum* infection. Individuals were classified as having incident symptomatic malaria if they were diagnosed with malaria at the study health facility in the 2 months preceding each survey. In addition, during routine visits individuals answered the yes/no question “Have you had malaria in the last two months?” and those individuals who answered yes were classified as having had an incident symptomatic episode even if they were not captured at the health facility. Incident asymptomatic infections were defined as parasite positive routine visits (with a parasite density exceeding 100 parasites/µL by qPCR) that were preceded by at least 2 prior visits that were negative by qPCR [15]. Individuals who were persistently qPCR positive and did not have intervening qPCR-negative visits were classified as having prevalent infection and did not contribute incident events under our outcome definition.

#### Covariates

We adjusted models for covariates that may influence both the likelihood of travel and malaria Models adjusted for potential confounders influencing both travel and malaria risk: (1) gender; (2) age (<5, 5–15, >15 years); (3) improved walls (burnt bricks, cement blocks, stone, timber, or iron sheets vs thatch, mud, unburnt bricks); (4) improved floors (parquet, tiles, bricks, cement/concrete, or stone vs earth, sand, dung); (5) household size; and (6) follow-up number (indicator) to account for seasonal variation in travel and malaria risk.

### Data Analysis

We used multilevel logistic regression models to estimate odd ratios (ORs) and 95% confidence intervals (CIs) for the relationship between travel variables and incident malaria. Our primary analysis included models for individuals living in all villages and models stratified by transmission intensity (high- vs low-transmission villages). Transmission intensity was defined using village-level prevalence of parasitemia at baseline measured via qPCR. High-transmission villages were defined as those with a prevalence of at least 10% (the median prevalence across all villages). Additionally, we assessed whether the associations between travel and incident malaria changed over time and further examined whether these associations varied by both time and village-level transmission intensity. For models including all villages, we incorporated village- and individual-level random effects. For models stratified by transmission intensity, we used individual-level random effects only. As a secondary analysis, we varied the threshold used to classify the transmission intensity (high vs low) using thresholds ranging from 5% to 50%. For this analysis, we allowed the classification of each village to vary over time based on village-level prevalence measured throughout follow-up visits every 2 months. This analysis estimated the association between travel-related variables and incident malaria across villages above and below each threshold.

We conducted sensitivity analyses considering only confirmed malaria cases detected at the health facility and asymptomatic infections as outcomes; these analyses excluded baseline data because we could not confirm cases prior to enrollment in the study. We conducted an additional sensitivity analysis that redefined the travel variables as self-reported travel occurring prior to the reported malaria episode date. For health facility-identified cases, this date corresponded to the facility record, while for cases ascertained during household survey visits, it was based on self-reported symptom onset. Both the timing of travel and the timing of malaria episodes in survey-identified cases were self-reported, so this restriction helped ensure that only travel episodes plausibly preceding infection were considered.

We estimated population attributable fractions (PAFs) and their 95% CI using the formula $PAF=(P_{e\;}\times (RR-1)/P_{e\;}\times (RR-1)+1),$ where $P_{e\;}$ is the prevalence of the exposure in the population and RR is the relative risk of the travel-related exposure and incident malaria. Here, ORs represented the ratio of risk for these associations. We estimated PAFs overall, by transmission intensity, season, and village. The PAFs resulting from these analyses represent the impact of direct importation on malaria burden, not including any secondary infections that may result from transmission downstream of an imported case.

## RESULTS

### Study Population Characteristics

A total of 1918 individuals from 400 households were enrolled (Table 1). Slightly more than half were female, and half were aged >15 years. Among adults (≥18 years), farming was the most common occupation. About one-quarter of participants had no formal education, over half completed primary school, and the remainder had secondary or higher education. Of all participants, 784 lived in high-transmission villages and 1134 in low-transmission villages, with similar demographic distributions across both settings.

**Table 1.** Characteristics of Cohort Participants at Baseline (or First Time Surveyed), Overall and Stratified by Village-Level Transmission Intensity

	All Villages (n = 1918)	High-Transmission Villages^a^ (n = 784)	Low-Transmission Villages (n = 1134)
Female, % (n)	57.2 (1097)	58.9 (462)	56.0 (635)
Age, % (n)			
<5	15.0 (288)	16.3 (128)	14.1 (160)
5–15	35.1 (674)	36.1 (283)	34.5 (391)
>15	49.8 (956)	47.6 (373)	51.4 (583)
Occupation (among those greater than 18 y), % (n)			
Commerce	6.0 (49)	6.8 (21)	5.6 (28)
Farmer	76.4 (620)	83.2 (257)	72.2 (363)
Student	5.3 (43)	4.5 (14)	5.8 (29)
Childcare/homemaker	1.6 (13)	0.3 (1)	2.4 (12)
Other	10.5 (85)	5.2 (16)	13.7 (69)
Education level			
None	24.4 (468)	26.7 (209)	22.8 (259)
Primary	54.8 (1052)	54.3 (426)	55.2 (626)
Secondary+	20.5 (394)	18.8 (147)	21.8 (247)
Used net previous night, % (n)	62.0 (1190)	64.4 (730)	58.7 (460)
Improved walls, % (n)	18.1 (348)	18.0 (141)	18.3 (348)
Improved floors, % (n)	46.3 (888)	49.8 (565)	46.3 (888)
Household size, mean (SD)	5.3 (2.1)	5.7 (2.3)	4.9 (1.8)

^a^Villages were classified as high transmission if baseline prevalence of *P. falciparum* infection in all ages was >10%.

### Travel Among Study Participants

Participants were followed for an average of 10.3 months (Table 2). At each visit, 8.2% reported overnight travel in the prior 2 months; over the full study, nearly one-third traveled at least once. Travel was more common among those over 15 (11.8%) than among children under 5 (7.2%) or aged 5–15 (3.4%) and slightly more common among males (9.0%) than females (6.7%). Trips averaged 1.7 weeks (range: 1–62 days), with 97.0% reporting just one trip per interval. Nearly half of trips were short-distance (<25 km from home), as shown in Supplementary Figure 2 and Supplementary Table 1. Most travel (55%) occurred outside the district but within Uganda, and 0.9% was international. Visiting relatives or friends was the predominant reason for travel.

**Table 2.** Description of Malaria and Travel Patterns Among Study Participants, Overall and Stratified by Village-level Transmission Intensity

	All Villages	High-Transmission Villages	Low-Transmission Villages
Number of participants, n	1918	784	1134
Number of follow-up months, mean (SD)	10.3 (4.1)	10.4 (4.2)	10.2 (4.1)
Malaria episodes, n	244	208	36
Incident asymptomatic *P. falciparum* episodes, n	45	34	11
Any incident infection^a^, n	283	237	45
Any self-reported overnight travel outside of village over study period, %	29.4	32.3	27.3
Any self-reported overnight travel outside of village in prior 2 months, %	8.2	8.9	7.6
Travel to higher incidence area in prior 2 months, %	4.9	5.2	4.6
Travel duration in weeks among travelers, mean (SD)	1.7 (2.4)	1.5 (2.3)	2.2 (2.5)
Travel destination among travelers, %			
Rukiga	35.6	44.1	28.8
Kampala	14.4	14.2	14.6
Rwanda	21.5	16.6	25.5
Other	28.5	25.1	31.1
Travel distance among trips, %			
Less than 25 km	47.8	53.0	43.6
25 to less than 50 km	25.0	24.0	25.8
Greater than 50 km	27.2	23.0	30.6
Travel reason among trips, %			
Visiting relatives/friends	65.7	69.3	62.8
School	9.2	4.2	13.3
Work/trading	10.3	10.8	9.9
Other	14.8	15.8	14.0

^a^“Any incident infection” includes incident symptomatic malaria episodes (identified at the health facility or by self-report) and incident asymptomatic infections detected by qPCR.

### Malaria Burden

During the study, 283 *P. falciparum* infections (symptomatic or asymptomatic) were detected among cohort participants, 237 of which occurred in high-transmission villages. Malaria incidence varied markedly over time (Figure 1*B*), peaking during the first 5 months before declining. In high-transmission villages (baseline prevalence ≥ 10%), median facility-based incidence was 331 cases per 1000 person-years (PY; IQR 96–536) initially, dropping nearly 10-fold to 39 per 1000 PY (IQR 17–60). In low-transmission villages, incidence decreased from 41 (IQR 22–74) to 12 per 1000 PY (IQR 0–25). Parasite prevalence showed parallel trends (Supplementary Figure 3).

### Associations Between Overnight Travel and Malaria by Transmission Intensity

Associations between overnight travel and malaria varied by transmission setting. Overall, any overnight travel increased malaria risk (OR = 1.68, 95% CI 1.06–2.66), with the effect concentrated in low-transmission villages (OR = 2.96, 95% CI 1.32–6.62), while no significant association was observed in high-transmission villages (OR = 1.22, 95% CI .75–1.99; Model 1 in Table 3; Supplementary Tables 2–6). Since travel-associated malaria may be more likely to occur when individuals travel to places with higher transmission than their home village, we then evaluated the association between overnight travels specifically to areas of higher incidence. Associations were stronger overall (Model 2, OR = 2.18, 95% CI 1.24–3.83) and in low-transmission villages (OR = 4.38, 95% CI 1.8–10.64), but not in high-transmission villages (OR = 1.62, 95% CI .94–2.8). Travel to higher incidence areas may directly account for 5.4% of malaria cases overall and 13.7% of cases in low-transmission villages. We also examined the role of trip duration. Compared to no travel, in low-transmission villages, long trips (≥1 week) were significantly associated with malaria (OR = 4.1, 95% CI 1.41–11.91). Finally, we assessed travel destination (Models 4 and 5). Trips within Rukiga District (where Kamwezi is located) were associated with increased malaria risk, while trips to other destinations were not. Travel to destinations within 25 km of the village was associated with greater odds of malaria, whereas longer trips were not associated. Trips within Rukiga accounted for 3.7% of cases overall and 7.8% of cases in low-transmission villages, suggesting local travel accounted for more than two-thirds of imported cases.

**Table 3.** Odds Ratios From Adjusted Models Assessing the Association Between Travel Variables and Incident Malaria Among Cohort Members

		All Villages	High-Transmission Villages	Low-Transmission Villages
	Exposure	OR (95% CI)	OR (95% CI)	OR (95% CI)
Model 1	Any overnight travel	1.68* (1.06, 2.66)	1.22 (.75, 1.99)	2.96** (1.32, 6.62)
Model 2	Travel to higher incidence area	2.18* (1.24, 3.83)	1.62^a^ (.94, 2.8)	4.38** (1.8, 10.64)
Model 3	Travel duration			
	No travel	Ref.	Ref.	Ref.
	Less than 1 week	1.78^a^ (.97, 3.24)	1.44 (.84, 2.47)	1.99 (.6, 6.63)
	One week or greater	1.53 (.55, 4.26)	.85 (.34, 2.08)	4.1* (1.41, 11.91)
Model 4	Travel destination			
	No travel	Ref.	Ref.	Ref.
	Rukiga	3.41* (1.17, 9.92)	2.58** (1.3, 5.13)	7.28** (2.18, 24.32)
	Other	1.44 (.48, 4.33)	.94 (.39, 2.27)	3.37^a^ (.99, 11.47)
Model 5	Travel distance			
	No travel	Ref.	Ref.	Ref.
	Less than 25 km	3.41^a^ (.8, 14.45)	2.58** (1.3, 5.13)	7.27** (2.17, 24.3)
	25 to less than 50 km	1.52 (.23, 9.96)	1.16 (.3, 4.47)	2.85 (.39, 20.84)
	50 km and more	1.4 (.19, 10.34)	.83 (.26, 2.64)	3.59^a^ (.8, 16.05)

Observations are from 1918 participants from a total of 6 visits. Adjusted for sex, age category, household improved floors, household improved walls, household size, and follow-up.

^a^< .1, *< .05, **< .01.

We repeated analyses limiting the definition of incident infections to laboratory-confirmed malaria cases diagnosed at the health facility and asymptomatic infections for the outcome. This analysis included 8255 observations of 1816 individuals, with 151 infections, 111 of which were laboratory-confirmed symptomatic incident malaria cases (two-thirds of the 186 cases captured during follow-ups that included self-reported cases). Of these 111 cases, 61 (55%) were accurately self-reported at the subsequent follow-up. With each additional week between the survey date and the facility visit, the probability of accurate self-reporting declined by 3 percentage points (95% CI: −6.4, −.1). In these analyses, we found consistent associations between travel and malaria overall, but not in low-transmission villages (Supplementary Tables 7 and 8), likely reflecting few cases in low-transmission villages (n = 24). Furthermore, we found consistent yet attenuated results when redefining travel as self-reported travel that occurred prior to the self-reported malaria case (Supplementary Table 9).

**Figure 2.**
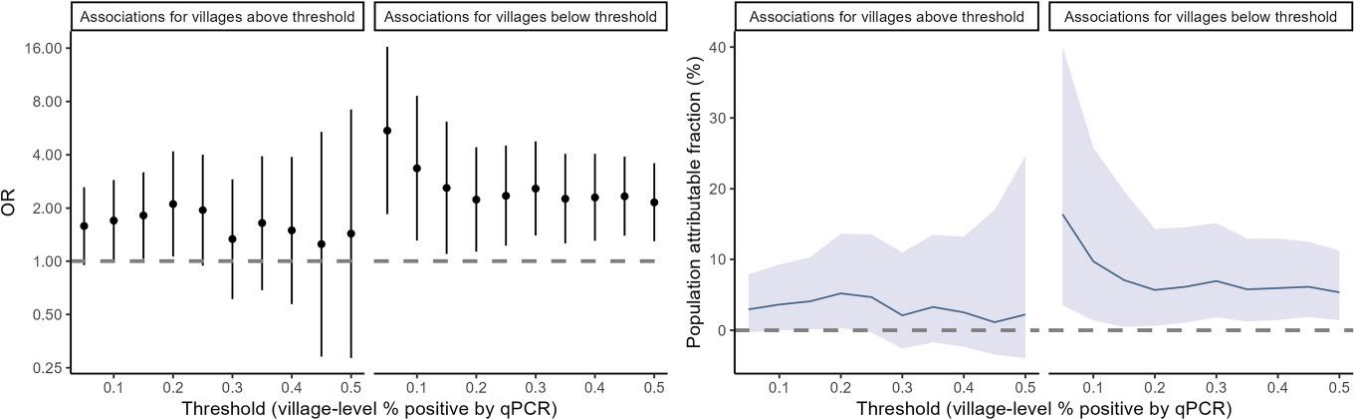
Relationships between travel to villages of higher incidence (ORs and 95% CI) and population attributable fractions when using varying thresholds to define high- versus and low-transmission villages.

To explore how definitions of “high” versus “low” transmission impact associations, we repeated analyses using varying thresholds and time-varying village-level prevalence (Figure 2). Travel was consistently associated with malaria across all definitions of low transmission, with stronger associations at lower thresholds. Overnight travel accounted for 11.1% of cases in villages with ≤20% prevalence and 16.4% in villages with <5%. In high-transmission villages, travel to higher incidence areas was associated with malaria only when lower thresholds were used (ie, including lower transmission villages), but not at thresholds ≥ 25%.

### Associations Between Travel and Malaria Over Time

**Figure 3.**
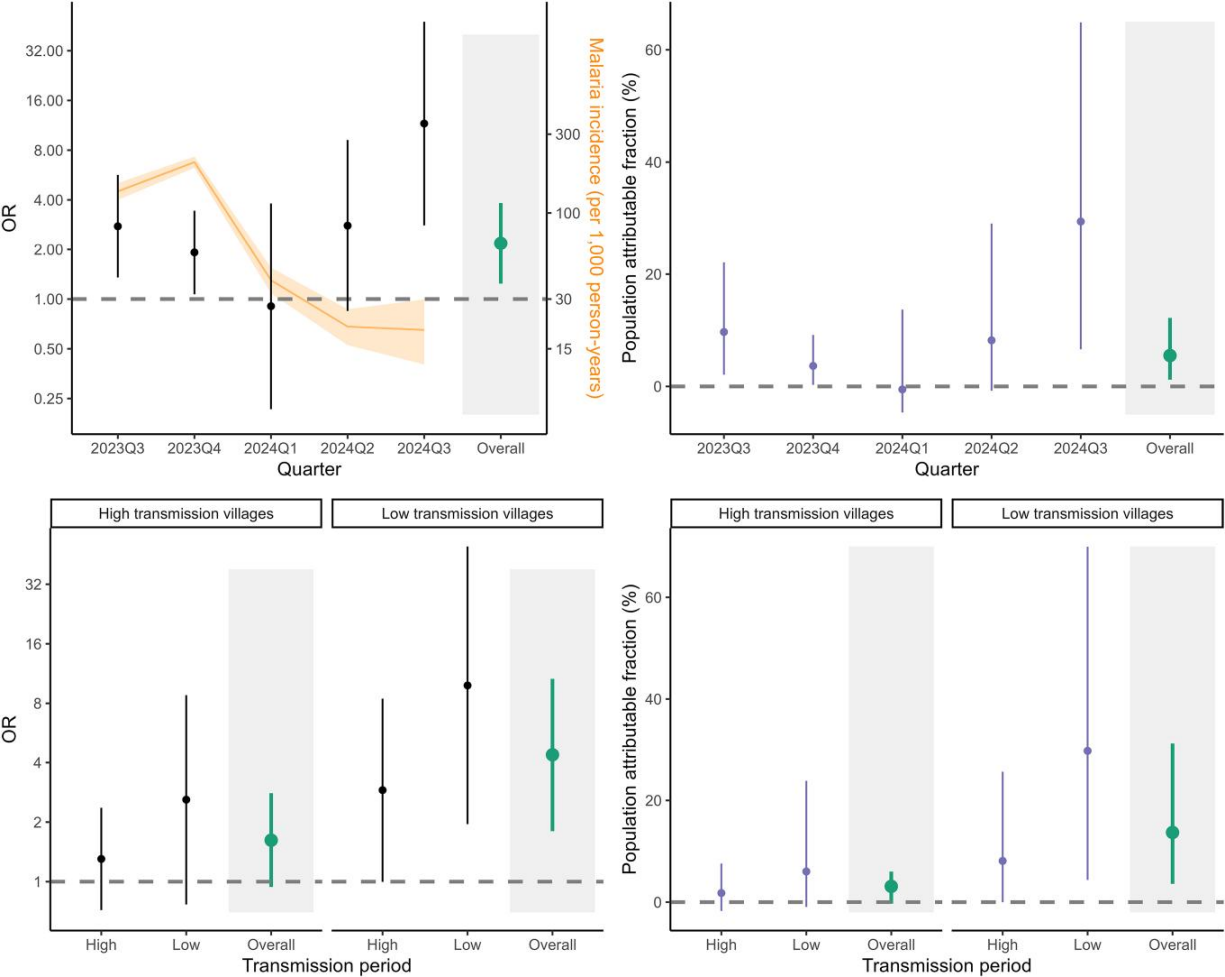
Relationship between travel to areas of higher incidence (ORs and 95% CI) and population attributable fractions over time and by time and village-level transmission intensity. Malaria incidence (and 95% CI) is shown in orange.

As malaria burden declined, the relationship between travel and infection strengthened. Across the full study area, travel to higher-transmission regions was most strongly associated with malaria during periods of low local transmission, accounting for 30% of cases in the third quarter of 2024 (Figure 3). In high-transmission villages, travel was not associated with malaria before 1 March 2024; while the association increased in low-transmission times, we could not rule out a null or negative association. In low-transmission villages, travel was consistently associated with malaria, accounting for 8.1% of cases during high transmission and 29.8% during low transmission. These patterns held when restricting to health facility-confirmed cases (Supplementary Figures 4 and 5).

### Heterogeneity in Malaria Attributable to Overnight Travel Over Space and Time

**Figure 4.**
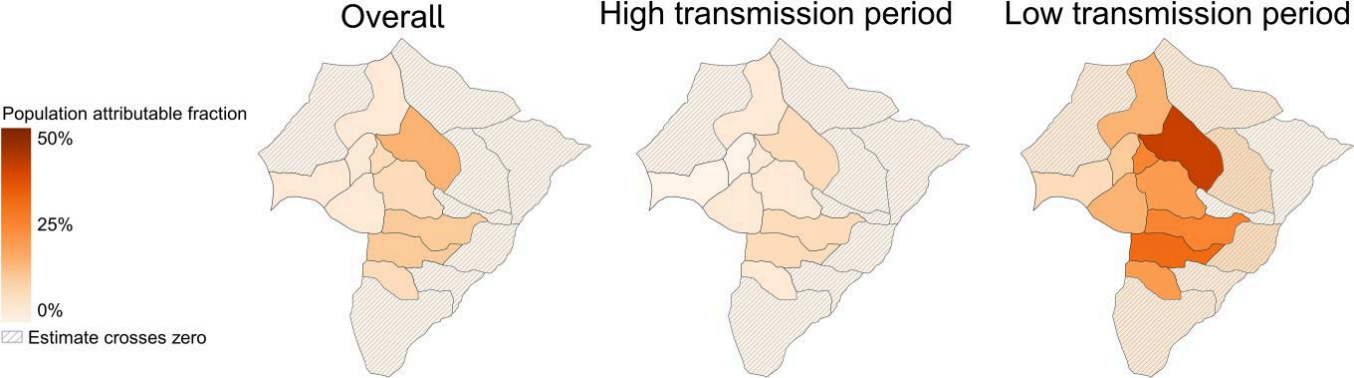
Village-level population attributable fractions representing the contribution of travel to higher incidence areas on malaria over the entire study period, during the high-transmission period, and during the low-transmission period.

Given variation in travel-malaria associations across space and time, we estimated PAFs by village and transmission period (Figure 4). Over the full study, travel accounted for a median of 5.9% (IQR 2.8%–11.6%) of cases. During high transmission, travel accounted for a median of 7.3% (IQR 5.5%–9.7%) in low-transmission villages; estimates in high-transmission villages were not significant. During low transmission, travel accounted for a median of 26.9% of cases in low-transmission villages (IQR 21.5%–33.3%), reaching up to 45% in some villages; estimates in high-transmission villages were again not significant. Population attributable fractions by sociodemographic variables appear in Supplementary Table 10.

## DISCUSSION

We evaluated the role of importation in a region of Uganda with spatial and temporal variation in transmission. While overall associations between travel and malaria were modest, travel was strongly linked to infection in low-transmission villages and during periods of reduced local transmission. These findings suggest that travel to higher-transmission areas contributes to malaria burden where local transmission alone cannot explain cases and that importation becomes important as local transmission declines.

These results fit the lens of receptivity and vulnerability, 2 concepts defining elimination potential [7, 16]. Receptivity in our study area is constrained by altitude and has been further reduced by high LLIN coverage and declining local prevalence. At the same time, vulnerability is high: frequent short-distance travel connects this low-receptivity area to neighboring higher-transmission settings. Moreover, in areas of low transmission and as transmission intensity declines, residents likely have reduced immunity. The interaction between declining receptivity and persistent vulnerability helps explain why travel became a strong driver of malaria risk in low-transmission villages and during periods of reduced local transmission.

The impact of importation varied across space and time. Although most prior studies focus on near-elimination settings [17–20], our study area had low-to-moderate incidence with substantial village-level and seasonal heterogeneity. Even in this context, importation became more important when local exposure declined. These findings align with a 2020 study in Zambia, where importation's importance increased during low-transmission periods but diminished when local exposure was high [21]. Together, the evidence suggests that importation's contribution to burden is dynamic and contingent on shifts in local transmission context.

While we estimate that travel may directly account for up to 45% of malaria cases in some of the study villages, it is important to note that the estimates of attribution derived from this study likely represent lower bounds of the true contribution of importation on malaria burden. Our estimates capture only the direct effect of overnight travel on incidence, without accounting for subsequent secondary cases that may arise from imported infections. Future work will combine these findings with genomic parasite data to estimate transmission chains and quantify the impact of importation on downstream cases. Furthermore, we do not account for additional potential sources of imported cases, including visitors [18, 22] and day trips. While these factors are harder to measure, they may also be contributors to burden.

Travel is a well-established malaria risk factor across Africa [23–27]. Here, both the occurrence and characteristics of travel mattered. Local mobility, rather than long-distance travel, was more predictive of risk and may account for two-thirds of imported cases in this setting. This has practical implications: malaria prevention efforts should not exclusively target international or cross-country travelers but also address the much more common pattern of local mobility. Longer duration trips were associated with greater risk of importation, a finding reflected in other settings [21, 28].

While there has long been an emphasis on the importance of importation in low transmission, near-elimination settings [3], our results indicate that it may be equally important in regions with moderate burden that have seen recent declines in transmission, as illustrated by our high-transmission villages [29]. Notably, the most recent universal distribution of LLINs in the study area occurred in November 2023, coinciding with a decline in malaria incidence. These LLINs included those treated with chlorfenapyr and alpha-cypermethrin, which have demonstrated high efficacy [30]. This timing raises the possibility that reduced local transmission following net distribution may have increased the relative contribution of travel to malaria burden. Although the LLIN distribution occurred in 2023 nationwide and was associated with reductions across large geographic areas, malaria incidence remained heterogeneous, with some nearby districts experiencing higher transmission than our study area even after the campaign. Thus, although the campaign reduced risk broadly, travel continued to link populations with differing levels of transmission intensity, sustaining opportunities for importation.

This finding has important public health implications. National Malaria Control Programs may consider a hybrid approach, combining interventions for locally acquired (eg, LLINs, IRS) and imported infections (eg, traveler screening or treatment). Notably, self-reported LLIN use in the cohort was relatively high (80% of participants reported using LLINs the night before the survey), but only 40% of travelers reported using a net while traveling. Future interventions may also aim to increase LLIN use among travelers. Implementation science research is needed to refine strategies targeting importation, ensuring that interventions are appropriate, feasible, and sustainable within health systems.

Strengths of this study include a well-characterized cohort with repeated measures and mobility data, enabling high-resolution spatial and temporal analyses. Combining household follow-up with health facility surveillance improved case detection and attribution. However, there are limitations. Reliance on self-reported malaria and travel histories introduces potential misclassification; we confirmed cases with facility records when possible and expect non-differential reporting, bias would likely be toward the null. Sensitivity analyses using confirmed cases were constrained by small samples in low-transmission settings, potentially limiting power. We also used external incidence estimates to classify destination transmission intensity, which may not fully capture local or seasonal variation. Future studies could improve on these aspects using spatial data and serological or genomic tools to validate exposure and infection.

Importation represents a dynamic and substantial contributor to malaria burden even outside elimination contexts. This underscores the need to move beyond a binary framework of control versus elimination and consider adopting a nuanced approach that recognizes the evolving role of importation across transmission settings. Ministries of Health, including those in high burden countries like Uganda, may consider implementing integrated strategies that address locally acquired and imported infections.

## Supplementary Material

jiag008_Supplementary_Data

## Supplementary Data

Supplementary materials are available at *The Journal of Infectious Diseases* online (http://jid.oxfordjournals.org/). Supplementary materials consist of data provided by the author that are published to benefit the reader. The posted materials are not copyedited. The contents of all supplementary data are the sole responsibility of the authors. Questions or messages regarding errors should be addressed to the author.

## References

[1] WHO . World malaria report 2024. Geneva: World Health Organization, 2024.

[2] Wesolowski A, Eagle N, Tatem AJ, et al Quantifying the impact of human mobility on malaria. Science 2012; 338:267–70. 23066082 10.1126/science.1223467 PMC3675794

[3] Sturrock HJW, Roberts KW, Wegbreit J, Ohrt C, Gosling RD. Tackling imported malaria: an elimination endgame. Am J Trop Med Hyg 2015; 93:139–44. 26013369 10.4269/ajtmh.14-0256 PMC4497886

[4] Martens P, Hall L. Malaria on the move: human population movement and malaria transmission. Emerg Infect Dis 2000; 6:103–9. 10756143 10.3201/eid0602.000202 PMC2640853

[5] Stoddard ST, Morrison AC, Vazquez-Prokopec GM, et al The role of human movement in the transmission of vector-borne pathogens. PLoS Negl Trop Dis 2009; 3:e481. 19621090 10.1371/journal.pntd.0000481 PMC2710008

[6] Ahmed S, Reithinger R, Kaptoge SK, Ngondi JM. Travel is a key risk factor for malaria transmission in pre-elimination settings in sub-saharan Africa: a review of the literature and meta-analysis. Am J Trop Med Hyg 2020; 103:1380–7. 32815497 10.4269/ajtmh.18-0456 PMC7543864

[7] Yukich JO, Lindblade K, Kolaczinski J. Receptivity to malaria: meaning and measurement. Malar J 2022; 21:145. 35527264 10.1186/s12936-022-04155-0 PMC9080212

[8] Uganda National Malaria Control Division (NMCD), Uganda Bureau of Statistics (UBOS), ICF . Uganda malaria indicator survey 2018–19. Kampala, Uganda, and Rockville, Maryland, USA: NMCD, UBOS, and ICF, 2020.

[9] National Malaria Control Programme . The Uganda malaria reduction and elimination strategic plan 2021–2025. Kampala: Ministry of Health Uganda, 2020.

[10] Talisuna AO, Noor AM, Okui AP, Snow RW. The past, present and future use of epidemiological intelligence to plan malaria vector control and parasite prevention in Uganda. Malar J 2015; 14:158. 25888989 10.1186/s12936-015-0677-4 PMC4399081

[11] Zalwango MG, Zalwango JF, Kadobera D, et al Evaluation of malaria outbreak detection methods, Uganda, 2022. Malar J 2024; 23:18. 38218860 10.1186/s12936-024-04838-w PMC10787982

[12] Teyssier NB, Chen A, Duarte EM, Sit R, Greenhouse B, Tessema SK. Optimization of whole-genome sequencing of Plasmodium falciparum from low-density dried blood spot samples. Malar J 2021; 20:116. 33637093 10.1186/s12936-021-03630-4 PMC7912882

[13] Hofmann N, Mwingira F, Shekalaghe S, Robinson LJ, Mueller I, Felger I. Ultra-sensitive detection of Plasmodium falciparum by amplification of multi-copy subtelomeric targets. PLoS Med 2015; 12:e1001788. 25734259 10.1371/journal.pmed.1001788 PMC4348198

[14] Weiss DJ, Lucas TCD, Nguyen M, et al Mapping the global prevalence, incidence, and mortality of Plasmodium falciparum, 2000–17: a spatial and temporal modelling study. Lancet 2019; 394:322–31. 31229234 10.1016/S0140-6736(19)31097-9 PMC6675740

[15] Briggs J, Teyssier N, Nankabirwa JI, et al Sex-based differences in clearance of chronic Plasmodium falciparum infection. Elife 2020; 9:e59872. 33107430 10.7554/eLife.59872 PMC7591246

[16] WHO . Global technical strategy for malaria 2016–2030. Geneva: World Health Organization, 2015.

[17] Guerra CA, Kang SY, Citron DT, et al Human mobility patterns and malaria importation on Bioko Island. Nat Commun 2019; 10:2332. 31133635 10.1038/s41467-019-10339-1 PMC6536527

[18] Le Menach A, Tatem AJ, Cohen JM, et al Travel risk, malaria importation and malaria transmission in Zanzibar. Sci Rep 2011; 1:93. 22355611 10.1038/srep00093 PMC3216579

[19] Wangdi K, Unwin HJT, Penjor K, et al Estimating the impact of imported malaria on local transmission in a near elimination setting: a case study from Bhutan. Lancet Reg Health Southeast Asia 2024; 31:100497. 39492850 10.1016/j.lansea.2024.100497 PMC11530917

[20] Dharmawardena P, Premaratne RG, Gunasekera WM, Hewawitarane M, Mendis K, Fernando D. Characterization of imported malaria, the largest threat to sustained malaria elimination from Sri Lanka. Malar J 2015; 14:177. 25902716 10.1186/s12936-015-0697-0 PMC4411700

[21] Porter TR, Finn TP, Silumbe K, et al Recent travel history and Plasmodium falciparum malaria infection in a region of heterogenous transmission in Southern Province, Zambia. Am J Trop Med Hyg 2020; 103:74–81. 32618250 10.4269/ajtmh.19-0660 PMC7416974

[22] Cosner C, Beier JC, Cantrell RS, et al The effects of human movement on the persistence of vector-borne diseases. J Theor Biol 2009; 258:550–60. 19265711 10.1016/j.jtbi.2009.02.016 PMC2684576

[23] Arinaitwe E, Dorsey G, Nankabirwa JI, et al Association between recent overnight travel and risk of malaria: a prospective cohort study at 3 sites in Uganda. Clin Infect Dis 2019; 68:313–20. 29868722 10.1093/cid/ciy478 PMC6321857

[24] Yukich JO, Taylor C, Eisele TP, et al Travel history and malaria infection risk in a low-transmission setting in Ethiopia: a case control study. Malar J 2013; 12:33. 23347703 10.1186/1475-2875-12-33 PMC3570338

[25] Chirebvu E, Chimbari MJ, Ngwenya BN. Assessment of risk factors associated with malaria transmission in Tubu village, Northern Botswana. Malar Res Treat 2014; 2014:403069. 24757573 10.1155/2014/403069 PMC3976786

[26] Mathanga DP, Tembo AK, Mzilahowa T, et al Patterns and determinants of malaria risk in urban and peri-urban areas of Blantyre, Malawi. Malar J 2016; 15:590. 27931234 10.1186/s12936-016-1623-9 PMC5146950

[27] Lynch CA, Bruce J, Bhasin A, Roper C, Cox J, Abeku TA. Association between recent internal travel and malaria in Ugandan highland and highland fringe areas. Trop Med Int Health 2015; 20:773–80. 25689689 10.1111/tmi.12480 PMC5006858

[28] Marshall JM, Toure M, Ouedraogo AL, et al Key traveller groups of relevance to spatial malaria transmission: a survey of movement patterns in four sub-Saharan African countries. Malar J 2016; 15:200. 27068686 10.1186/s12936-016-1252-3 PMC4828820

[29] Hergott DEB, Guerra CA, Garcia GA, et al Impact of six-month COVID-19 travel moratorium on Plasmodium falciparum prevalence on Bioko Island, Equatorial Guinea. Nat Commun 2024; 15:8285. 39333562 10.1038/s41467-024-52638-2 PMC11436818

[30] Mosha JF, Kulkarni MA, Lukole E, et al Effectiveness and cost-effectiveness against malaria of three types of dual-active-ingredient long-lasting insecticidal nets (LLINs) compared with pyrethroid-only LLINs in Tanzania: a four-arm, cluster-randomised trial. Lancet 2022; 399:1227–41. 35339225 10.1016/S0140-6736(21)02499-5 PMC8971961

