# Supplementary material for: Quantifying the Role of Importation on Sustained Malaria Transmission in a Low-to-Moderate Burden Region of Southwest Uganda
Source: J Infect Dis. 2026 Jan 6;233(4):e872–81. doi: 10.1093/infdis/jiag008 (PMC13127743; doi:10.1093/infdis/jiag008)
Supplement: jiag008_Supplementary_Data [file jiag008_supplementary_data.docx]

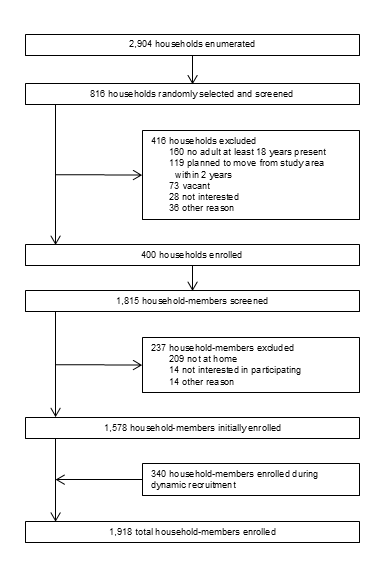


**Supplemental Figure 1.** Enrollment and follow-up of study participants.


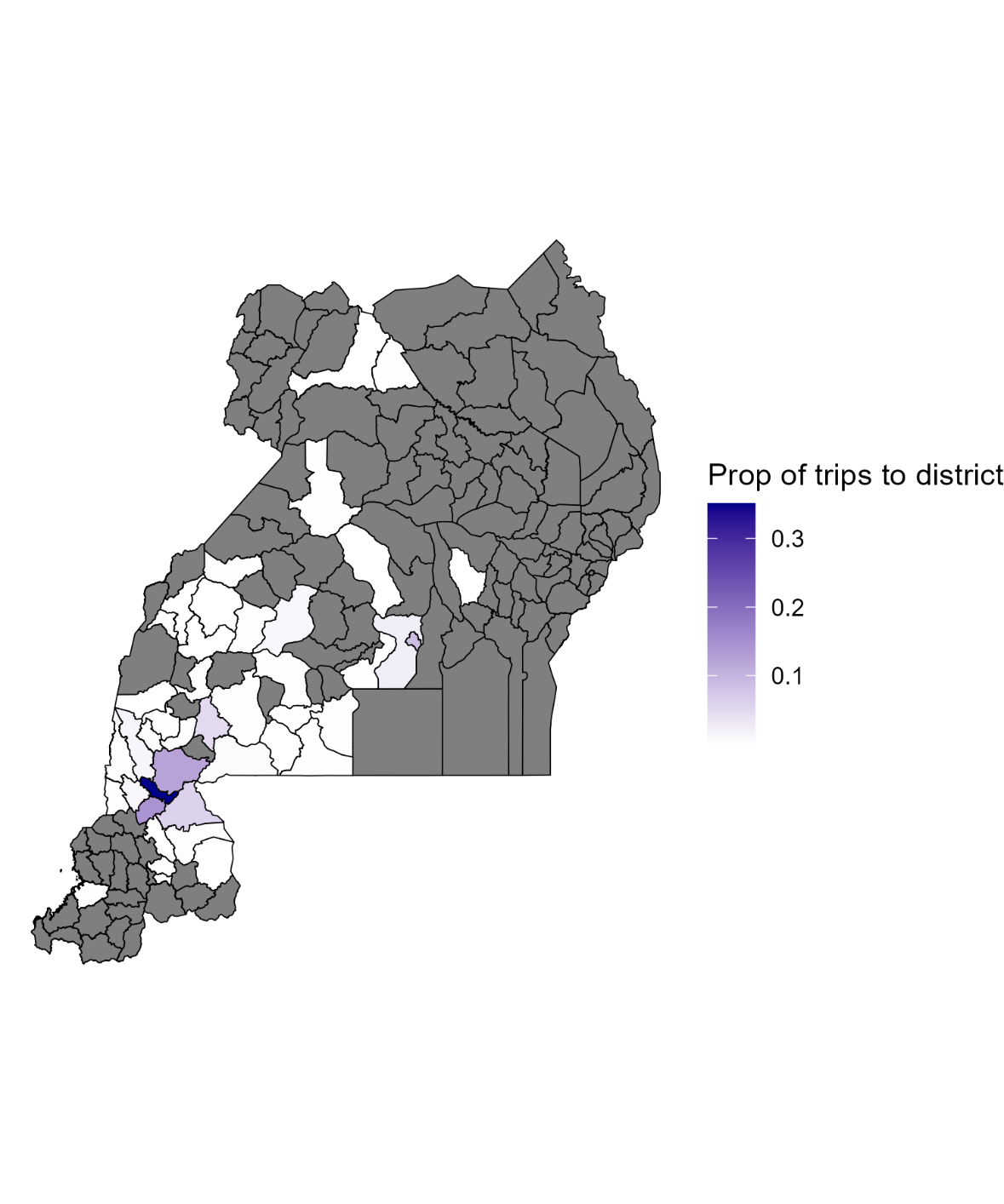


**Supplemental Figure 2**. Proportion of trips at the district-level among cohort members.


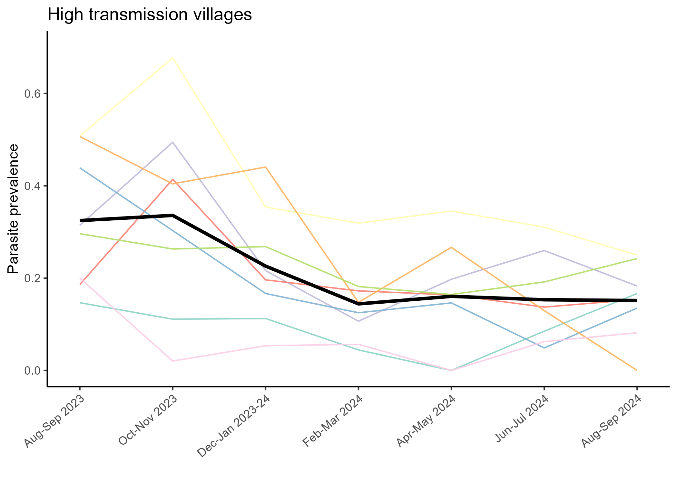

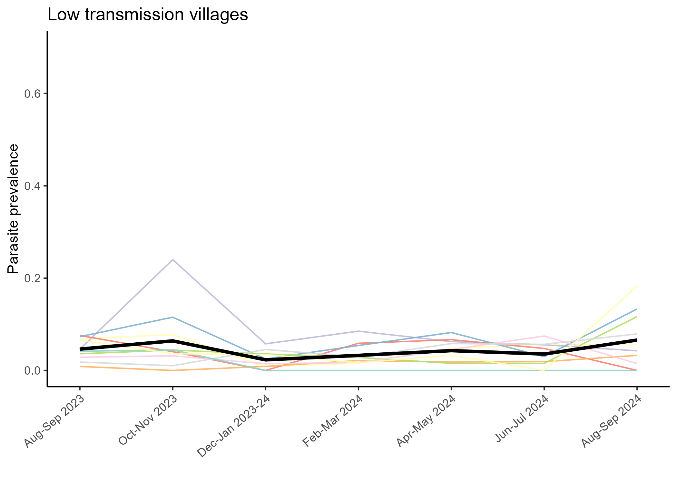


**Supplemental Figure 3.** Prevalence of *Plasmodium falciparum* over time stratified by village-level transmission intensity, presented as averages (black lines) and by village (colored lines).

| **Supplemental Table 1.** Destinations of trips among cohort members. | | | | |
| --- | --- | --- | --- | --- |
| District | Country | Proportion of trips | Count of trips | Malaria incidence (per 1,000)* |
| RUKIGA | Uganda | 0.351 | 254 | 93.0 |
| KABALE | Uganda | 0.145 | 105 | 5.9 |
| NTUNGAMO | Uganda | 0.123 | 89 | 141.4 |
| KAMPALA | Uganda | 0.087 | 63 | 150.6 |
| NYAGATARE | Rwanda | 0.059 | 43 | 77.9 |
| MBARARA | Uganda | 0.050 | 36 | 170.3 |
| WAKISO | Uganda | 0.024 | 17 | 195.5 |
| RUKUNGIRI | Uganda | 0.015 | 11 | 179.6 |
| RUBANDA | Uganda | 0.014 | 10 | 3.7 |
| MUBENDE | Uganda | 0.011 | 8 | 226.3 |
| ISINGIRO | Uganda | 0.008 | 6 | 150.8 |
| MITOOMA | Uganda | 0.007 | 5 | 200.9 |
| KIRUHURA | Uganda | 0.006 | 4 | 203.5 |
| KYEGEGWA | Uganda | 0.006 | 4 | 227.4 |
| MASAKA | Uganda | 0.006 | 4 | 187.2 |
| GATSIBO | Rwanda | 0.006 | 4 | 77.4 |
| IBANDA | Uganda | 0.004 | 3 | 179.1 |
| KAGADI | Uganda | 0.004 | 3 | 234.0 |
| KANUNGU | Uganda | 0.004 | 3 | 166.2 |
| LWENGO | Uganda | 0.004 | 3 | 210.3 |
| SSEMBABULE | Uganda | 0.004 | 3 | 232.7 |
| KARONGI | Rwanda | 0.004 | 3 | 39.4 |
| BUSHENYI | Uganda | 0.003 | 2 | 191.4 |
| GULU | Uganda | 0.003 | 2 | 462.7 |
| KISORO | Uganda | 0.003 | 2 | 33.3 |
| KYOTERA | Uganda | 0.003 | 2 | 184.3 |
| MASINDI | Uganda | 0.003 | 2 | 256.2 |
| MPIGI | Uganda | 0.003 | 2 | 178.4 |
| GASABO | Rwanda | 0.003 | 2 | 70.5 |
| SHEEMA | Uganda | 0.003 | 2 | 176.8 |
| AMURU | Uganda | 0.001 | 1 | 396.4 |
| BUNYANGABU | Uganda | 0.001 | 1 | 139.3 |
| KABAROLE | Uganda | 0.001 | 1 | 185.4 |
| KAMULI | Uganda | 0.001 | 1 | 348.6 |
| KAMWENGE | Uganda | 0.001 | 1 | 196.2 |
| KYENJOJO | Uganda | 0.001 | 1 | 204.3 |
| NAKASEKE | Uganda | 0.001 | 1 | 239.4 |
| RAKAI | Uganda | 0.001 | 1 | 186.6 |
| KICUKIRO | Rwanda | 0.001 | 1 | 69.9 |
| RUBIRIZI | Uganda | 0.001 | 1 | 185.5 |
| KAYONZA | Rwanda | 0.001 | 1 | 80.8 |
| GICUMBI | Rwanda | 0.001 | 1 | 35.7 |
| *Estimated by Malaria Atlas Project | | | | |

####

| **Supplemental Table 2.** Odds ratios from adjusted models assessing the association between travel and incident malaria among cohort members. | | | | | | | | | |
| --- | --- | --- | --- | --- | --- | --- | --- | --- | --- |
|  | **All villages** | | | **High transmission villages** | | | **Low transmission villages** | | |
| **Characteristic** | **OR***^1^* | **95% CI***^1^* | **p-value** | **OR***^1^* | **95% CI***^1^* | **p-value** | **OR***^1^* | **95% CI***^1^* | **p-value** |
| Any overnight travel |  |  |  |  |  |  |  |  |  |
| No travel | — | — |  | — | — |  | — | — |  |
| Travel | 1.68 | 1.06, 2.66 | 0.036 | 1.22 | 0.75, 1.99 | 0.4 | 2.96 | 1.32, 6.62 | 0.008 |
| Male gender | 0.94 | 0.71, 1.24 | 0.6 | 1.01 | 0.74, 1.38 | >0.9 | 1.10 | 0.59, 2.06 | 0.8 |
| Age category |  |  |  |  |  |  |  |  |  |
| < 5 | — | — |  | — | — |  | — | — |  |
| 5-15 | 1.64 | 0.89, 3.05 | 0.089 | 1.54 | 1.04, 2.30 | 0.032 | 2.48 | 0.93, 6.66 | 0.070 |
| > 15 | 0.83 | 0.40, 1.71 | 0.5 | 0.81 | 0.53, 1.24 | 0.3 | 1.38 | 0.51, 3.77 | 0.5 |
| Improved walls | 0.75 | 0.34, 1.66 | 0.4 | 0.71 | 0.41, 1.22 | 0.2 | 0.76 | 0.29, 1.98 | 0.6 |
| Improved floor | 0.55 | 0.32, 0.95 | 0.038 | 0.59 | 0.40, 0.88 | 0.009 | 1.08 | 0.55, 2.13 | 0.8 |
| Household size | 1.16 | 0.99, 1.35 | 0.061 | 1.09 | 1.01, 1.18 | 0.020 | 0.92 | 0.76, 1.12 | 0.4 |
| Follow-up |  |  |  |  |  |  |  |  |  |
| 0 | — | — |  | — | — |  | — | — |  |
| 1 | 1.64 | 0.91, 2.95 | 0.079 | 1.71 | 1.18, 2.47 | 0.004 | 1.22 | 0.52, 2.83 | 0.6 |
| 2 | 1.46 | 0.85, 2.50 | 0.13 | 1.47 | 0.99, 2.17 | 0.056 | 1.51 | 0.66, 3.45 | 0.3 |
| 3 | 0.48 | 0.18, 1.26 | 0.10 | 0.44 | 0.26, 0.75 | 0.003 | 0.58 | 0.19, 1.71 | 0.3 |
| 4 | 0.44 | 0.26, 0.75 | 0.013 | 0.43 | 0.25, 0.74 | 0.002 | 0.45 | 0.14, 1.44 | 0.2 |
| 5 | 0.30 | 0.13, 0.70 | 0.016 | 0.26 | 0.13, 0.51 | <0.001 | 0.48 | 0.15, 1.54 | 0.2 |
| 6 | 0.12 | 0.05, 0.31 | 0.003 | 0.14 | 0.06, 0.32 | <0.001 | 0.00 | 0.00, 0.00 | <0.001 |
| *^1^*OR = Odds Ratio, CI = Confidence Interval | | | | | | | | | |

| **Supplemental Table 3.** Odds ratios from adjusted models assessing the association between travel to an area of higher incidence and incident malaria among cohort members. | | | | | | | | | |
| --- | --- | --- | --- | --- | --- | --- | --- | --- | --- |
|  | **All villages** | | | **High transmission villages** | | | **Low transmission villages** | | |
| **Characteristic** | **OR***^1^* | **95% CI***^1^* | **p-value** | **OR***^1^* | **95% CI***^1^* | **p-value** | **OR***^1^* | **95% CI***^1^* | **p-value** |
| Travel to higher incidence area |  |  |  |  |  |  |  |  |  |
| No travel | — | — |  | — | — |  | — | — |  |
| Travel | 2.18 | 1.24, 3.83 | 0.019 | 1.62 | 0.94, 2.80 | 0.082 | 4.38 | 1.80, 10.6 | 0.001 |
| Male gender | 0.94 | 0.71, 1.24 | 0.6 | 1.01 | 0.73, 1.38 | >0.9 | 1.09 | 0.58, 2.05 | 0.8 |
| Age category |  |  |  |  |  |  |  |  |  |
| < 5 | — | — |  | — | — |  | — | — |  |
| 5-15 | 1.65 | 0.88, 3.07 | 0.090 | 1.55 | 1.04, 2.31 | 0.030 | 2.55 | 0.95, 6.83 | 0.063 |
| > 15 | 0.83 | 0.40, 1.72 | 0.5 | 0.80 | 0.52, 1.23 | 0.3 | 1.42 | 0.53, 3.85 | 0.5 |
| Improved walls | 0.75 | 0.34, 1.63 | 0.4 | 0.71 | 0.41, 1.21 | 0.2 | 0.73 | 0.28, 1.92 | 0.5 |
| Improved floor | 0.56 | 0.32, 0.95 | 0.038 | 0.59 | 0.40, 0.87 | 0.009 | 1.11 | 0.56, 2.23 | 0.8 |
| Household size | 1.16 | 0.99, 1.35 | 0.063 | 1.09 | 1.01, 1.18 | 0.019 | 0.92 | 0.76, 1.12 | 0.4 |
| Follow-up |  |  |  |  |  |  |  |  |  |
| 0 | — | — |  | — | — |  | — | — |  |
| 1 | 1.64 | 0.91, 2.96 | 0.079 | 1.70 | 1.18, 2.46 | 0.005 | 1.24 | 0.53, 2.92 | 0.6 |
| 2 | 1.46 | 0.85, 2.52 | 0.13 | 1.47 | 0.99, 2.18 | 0.055 | 1.52 | 0.66, 3.51 | 0.3 |
| 3 | 0.48 | 0.18, 1.26 | 0.10 | 0.44 | 0.26, 0.75 | 0.003 | 0.58 | 0.19, 1.71 | 0.3 |
| 4 | 0.44 | 0.26, 0.75 | 0.013 | 0.43 | 0.25, 0.74 | 0.002 | 0.46 | 0.14, 1.47 | 0.2 |
| 5 | 0.30 | 0.13, 0.70 | 0.017 | 0.26 | 0.13, 0.51 | <0.001 | 0.48 | 0.15, 1.56 | 0.2 |
| 6 | 0.12 | 0.05, 0.31 | 0.003 | 0.14 | 0.06, 0.32 | <0.001 | 0.00 | 0.00, 0.00 | <0.001 |
| *^1^*OR = Odds Ratio, CI = Confidence Interval | | | | | | | | | |

####

| **Supplemental Table 4.** Odds ratios from adjusted models assessing the association between travel duration and incident malaria among cohort members. | | | | | | | | | |
| --- | --- | --- | --- | --- | --- | --- | --- | --- | --- |
|  | **All villages** | | | **High transmission villages** | | | **Low transmission villages** | | |
| **Characteristic** | **OR***^1^* | **95% CI***^1^* | **p-value** | **OR***^1^* | **95% CI***^1^* | **p-value** | **OR***^1^* | **95% CI***^1^* | **p-value** |
| Travel duration |  |  |  |  |  |  |  |  |  |
| No travel | — | — |  | — | — |  | — | — |  |
| Less than 1 week | 1.78 | 0.97, 3.24 | 0.056 | 1.44 | 0.84, 2.47 | 0.2 | 1.99 | 0.60, 6.63 | 0.3 |
| 1 week or greater | 1.53 | 0.55, 4.26 | 0.3 | 0.85 | 0.34, 2.08 | 0.7 | 4.10 | 1.41, 11.9 | 0.010 |
| Male gender | 0.94 | 0.68, 1.30 | 0.6 | 1.02 | 0.74, 1.39 | >0.9 | 1.09 | 0.58, 2.05 | 0.8 |
| Age category |  |  |  |  |  |  |  |  |  |
| < 5 | — | — |  | — | — |  | — | — |  |
| 5-15 | 1.65 | 0.81, 3.35 | 0.11 | 1.56 | 1.05, 2.32 | 0.030 | 2.43 | 0.90, 6.51 | 0.079 |
| > 15 | 0.83 | 0.36, 1.91 | 0.5 | 0.81 | 0.53, 1.24 | 0.3 | 1.40 | 0.51, 3.80 | 0.5 |
| Improved walls | 0.75 | 0.30, 1.86 | 0.4 | 0.70 | 0.41, 1.21 | 0.2 | 0.75 | 0.29, 1.97 | 0.6 |
| Improved floor | 0.55 | 0.30, 1.03 | 0.055 | 0.59 | 0.40, 0.88 | 0.009 | 1.06 | 0.54, 2.08 | 0.9 |
| Household size | 1.16 | 0.97, 1.38 | 0.081 | 1.09 | 1.01, 1.18 | 0.019 | 0.92 | 0.76, 1.12 | 0.4 |
| Follow-up |  |  |  |  |  |  |  |  |  |
| 0 | — | — |  | — | — |  | — | — |  |
| 1 | 1.64 | 0.84, 3.21 | 0.10 | 1.71 | 1.18, 2.47 | 0.005 | 1.19 | 0.51, 2.78 | 0.7 |
| 2 | 1.46 | 0.78, 2.73 | 0.15 | 1.47 | 0.99, 2.18 | 0.055 | 1.44 | 0.62, 3.37 | 0.4 |
| 3 | 0.48 | 0.16, 1.45 | 0.13 | 0.44 | 0.26, 0.75 | 0.003 | 0.56 | 0.19, 1.64 | 0.3 |
| 4 | 0.45 | 0.24, 0.81 | 0.023 | 0.43 | 0.25, 0.74 | 0.002 | 0.44 | 0.14, 1.41 | 0.2 |
| 5 | 0.30 | 0.12, 0.79 | 0.028 | 0.26 | 0.13, 0.51 | <0.001 | 0.46 | 0.14, 1.51 | 0.2 |
| 6 | 0.12 | 0.04, 0.35 | 0.008 | 0.14 | 0.06, 0.32 | <0.001 | 0.00 | 0.00, 0.00 | <0.001 |
| *^1^*OR = Odds Ratio, CI = Confidence Interval | | | | | | | | | |

####

| **Supplemental Table 5.** Odds ratios from adjusted models assessing the association between travel destination and incident malaria among cohort members. | | | | | | | | | |
| --- | --- | --- | --- | --- | --- | --- | --- | --- | --- |
|  | **All villages** | | | **High transmission villages** | | | **Low transmission villages** | | |
| **Characteristic** | **OR***^1^* | **95% CI***^1^* | **p-value** | **OR***^1^* | **95% CI***^1^* | **p-value** | **OR***^1^* | **95% CI***^1^* | **p-value** |
| Travel destination |  |  |  |  |  |  |  |  |  |
| No travel | — | — |  | — | — |  | — | — |  |
| RUKIGA | 3.41 | 1.17, 9.92 | 0.036 | 2.58 | 1.30, 5.13 | 0.007 | 7.28 | 2.18, 24.3 | 0.001 |
| Other | 1.44 | 0.48, 4.33 | 0.4 | 0.94 | 0.39, 2.27 | 0.9 | 3.37 | 0.99, 11.5 | 0.052 |
| Male gender | 0.94 | 0.68, 1.31 | 0.6 | 1.01 | 0.74, 1.39 | >0.9 | 1.10 | 0.58, 2.07 | 0.8 |
| Age category |  |  |  |  |  |  |  |  |  |
| < 5 | — | — |  | — | — |  | — | — |  |
| 5-15 | 1.66 | 0.81, 3.38 | 0.11 | 1.55 | 1.04, 2.31 | 0.031 | 2.61 | 0.98, 6.94 | 0.054 |
| > 15 | 0.83 | 0.36, 1.91 | 0.5 | 0.79 | 0.51, 1.22 | 0.3 | 1.46 | 0.56, 3.84 | 0.4 |
| Improved walls | 0.75 | 0.30, 1.84 | 0.4 | 0.71 | 0.41, 1.22 | 0.2 | 0.73 | 0.28, 1.89 | 0.5 |
| Improved floor | 0.56 | 0.30, 1.04 | 0.058 | 0.59 | 0.40, 0.88 | 0.009 | 1.15 | 0.57, 2.29 | 0.7 |
| Household size | 1.15 | 0.97, 1.38 | 0.084 | 1.09 | 1.01, 1.17 | 0.022 | 0.92 | 0.76, 1.12 | 0.4 |
| Follow-up |  |  |  |  |  |  |  |  |  |
| 0 | — | — |  | — | — |  | — | — |  |
| 1 | 1.63 | 0.83, 3.22 | 0.11 | 1.70 | 1.17, 2.45 | 0.005 | 1.24 | 0.52, 2.93 | 0.6 |
| 2 | 1.47 | 0.79, 2.76 | 0.14 | 1.48 | 1.00, 2.19 | 0.051 | 1.56 | 0.68, 3.59 | 0.3 |
| 3 | 0.48 | 0.16, 1.45 | 0.13 | 0.44 | 0.26, 0.75 | 0.003 | 0.58 | 0.20, 1.73 | 0.3 |
| 4 | 0.44 | 0.24, 0.80 | 0.023 | 0.43 | 0.25, 0.73 | 0.002 | 0.46 | 0.14, 1.48 | 0.2 |
| 5 | 0.31 | 0.12, 0.80 | 0.029 | 0.26 | 0.13, 0.52 | <0.001 | 0.50 | 0.16, 1.61 | 0.2 |
| 6 | 0.12 | 0.04, 0.35 | 0.008 | 0.14 | 0.06, 0.32 | <0.001 | 0.00 | 0.00, 0.00 | <0.001 |
| *^1^*OR = Odds Ratio, CI = Confidence Interval | | | | | | | | | |

####

| **Supplemental Table 6.** Odds ratios from adjusted models assessing the association between travel distance and incident malaria among cohort members. | | | | | | | | | |
| --- | --- | --- | --- | --- | --- | --- | --- | --- | --- |
|  | **All villages** | | | **High transmission villages** | | | **Low transmission villages** | | |
| **Characteristic** | **OR***^1^* | **95% CI***^1^* | **p-value** | **OR***^1^* | **95% CI***^1^* | **p-value** | **OR***^1^* | **95% CI***^1^* | **p-value** |
| Travel distance |  |  |  |  |  |  |  |  |  |
| No travel | — | — |  | — | — |  | — | — |  |
| Less than 25 km | 3.41 | 0.80, 14.4 | 0.068 | 2.58 | 1.30, 5.13 | 0.007 | 7.27 | 2.17, 24.3 | 0.001 |
| 25- <50km | 1.52 | 0.23, 9.96 | 0.4 | 1.16 | 0.30, 4.47 | 0.8 | 2.85 | 0.39, 20.8 | 0.3 |
| 50km + | 1.40 | 0.19, 10.3 | 0.5 | 0.83 | 0.26, 2.64 | 0.8 | 3.59 | 0.80, 16.0 | 0.095 |
| Male gender | 0.94 | 0.60, 1.47 | 0.6 | 1.01 | 0.74, 1.39 | >0.9 | 1.10 | 0.58, 2.07 | 0.8 |
| Age category |  |  |  |  |  |  |  |  |  |
| < 5 | — | — |  | — | — |  | — | — |  |
| 5-15 | 1.66 | 0.63, 4.37 | 0.2 | 1.55 | 1.04, 2.31 | 0.030 | 2.60 | 0.98, 6.93 | 0.056 |
| > 15 | 0.83 | 0.27, 2.58 | 0.6 | 0.79 | 0.52, 1.23 | 0.3 | 1.46 | 0.55, 3.83 | 0.4 |
| Improved walls | 0.75 | 0.22, 2.52 | 0.4 | 0.71 | 0.41, 1.22 | 0.2 | 0.73 | 0.28, 1.90 | 0.5 |
| Improved floor | 0.56 | 0.24, 1.29 | 0.10 | 0.59 | 0.40, 0.88 | 0.010 | 1.15 | 0.57, 2.29 | 0.7 |
| Household size | 1.15 | 0.91, 1.46 | 0.13 | 1.09 | 1.01, 1.17 | 0.022 | 0.92 | 0.76, 1.12 | 0.4 |
| Follow-up |  |  |  |  |  |  |  |  |  |
| 0 | — | — |  | — | — |  | — | — |  |
| 1 | 1.63 | 0.65, 4.10 | 0.15 | 1.69 | 1.17, 2.45 | 0.005 | 1.24 | 0.53, 2.90 | 0.6 |
| 2 | 1.47 | 0.63, 3.44 | 0.2 | 1.48 | 1.00, 2.19 | 0.051 | 1.56 | 0.68, 3.56 | 0.3 |
| 3 | 0.48 | 0.11, 2.15 | 0.2 | 0.44 | 0.26, 0.75 | 0.003 | 0.58 | 0.20, 1.70 | 0.3 |
| 4 | 0.44 | 0.19, 1.00 | 0.049 | 0.43 | 0.25, 0.73 | 0.002 | 0.45 | 0.14, 1.47 | 0.2 |
| 5 | 0.31 | 0.08, 1.12 | 0.059 | 0.26 | 0.13, 0.52 | <0.001 | 0.50 | 0.16, 1.61 | 0.2 |
| 6 | 0.12 | 0.03, 0.51 | 0.024 | 0.14 | 0.06, 0.32 | <0.001 | 0.00 | 0.00, 0.00 | <0.001 |
| *^1^*OR = Odds Ratio, CI = Confidence Interval | | | | | | | | | |

| **Supplemental Table 7.** Odds ratios from adjusted models assessing the association between travel and incident malaria among cohort members, with only confirmed symptomatic malaria cases and asymptomatic infections as outcome. | | | | | | | | | |
| --- | --- | --- | --- | --- | --- | --- | --- | --- | --- |
|  | **All villages** | | | **High transmission villages** | | | **Low transmission villages** | | |
| **Characteristic** | **OR***^1^* | **95% CI***^1^* | **p-value** | **OR***^1^* | **95% CI***^1^* | **p-value** | **OR***^1^* | **95% CI***^1^* | **p-value** |
| Any overnight travel |  |  |  |  |  |  |  |  |  |
| No travel | — | — |  | — | — |  | — | — |  |
| Travel | 1.68 | 0.93, 3.04 | 0.075 | 1.50 | 0.76, 2.96 | 0.2 | 1.89 | 0.46, 7.80 | 0.4 |
| Male gender | 0.87 | 0.58, 1.28 | 0.4 | 0.85 | 0.56, 1.29 | 0.4 | 1.58 | 0.69, 3.62 | 0.3 |
| Age category |  |  |  |  |  |  |  |  |  |
| < 5 | — | — |  | — | — |  | — | — |  |
| 5-15 | 2.74 | 1.56, 4.82 | 0.006 | 2.65 | 1.46, 4.82 | 0.001 | 5.60 | 0.72, 43.5 | 0.10 |
| > 15 | 1.39 | 0.84, 2.28 | 0.2 | 1.21 | 0.64, 2.28 | 0.6 | 5.32 | 0.72, 39.6 | 0.10 |
| Improved walls | 0.82 | 0.31, 2.17 | 0.6 | 0.70 | 0.32, 1.56 | 0.4 | 1.03 | 0.30, 3.52 | >0.9 |
| Improved floor | 0.46 | 0.24, 0.90 | 0.032 | 0.51 | 0.30, 0.87 | 0.014 | 0.79 | 0.31, 2.01 | 0.6 |
| Household size | 1.26 | 1.01, 1.56 | 0.041 | 1.20 | 1.08, 1.33 | <0.001 | 0.92 | 0.76, 1.11 | 0.4 |
| Follow-up |  |  |  |  |  |  |  |  |  |
| 0 | — | — |  | — | — |  | — | — |  |
| 1 | 2.34 | 1.18, 4.62 | 0.024 | 2.42 | 1.53, 3.83 | <0.001 | 2.93 | 0.88, 9.71 | 0.079 |
| 2 | 0.93 | 0.31, 2.85 | 0.9 | 0.85 | 0.48, 1.51 | 0.6 | 1.65 | 0.40, 6.85 | 0.5 |
| 3 | 0.75 | 0.38, 1.47 | 0.3 | 0.69 | 0.36, 1.31 | 0.3 | 1.29 | 0.30, 5.55 | 0.7 |
| 4 | 0.55 | 0.18, 1.66 | 0.2 | 0.50 | 0.24, 1.05 | 0.068 | 1.02 | 0.20, 5.13 | >0.9 |
| 5 | 0.16 | 0.04, 0.59 | 0.015 | 0.17 | 0.06, 0.51 | 0.001 | 0.00 | 0.00, 0.00 | <0.001 |
| 6 | 1.68 | 0.93, 3.04 | 0.075 | 1.50 | 0.76, 2.96 | 0.2 | 1.89 | 0.46, 7.80 | 0.4 |
| *^1^*OR = Odds Ratio, CI = Confidence Interval | | | | | | | | | |

| **Supplemental Table 8.** Odds ratios from adjusted models assessing the association between travel to an area of higher incidence and incident malaria among cohort members, with only confirmed symptomatic malaria cases and asymptomatic infections as outcome. | | | | | | | | | |
| --- | --- | --- | --- | --- | --- | --- | --- | --- | --- |
|  | **All villages** | | | **High transmission villages** | | | **Low transmission villages** | | |
| **Characteristic** | **OR***^1^* | **95% CI***^1^* | **p-value** | **OR***^1^* | **95% CI***^1^* | **p-value** | **OR***^1^* | **95% CI***^1^* | **p-value** |
| Travel to higher incidence area |  |  |  |  |  |  |  |  |  |
| No travel | — | — |  | — | — |  | — | — |  |
| Travel | 2.13 | 1.09, 4.17 | 0.033 | 1.82 | 0.86, 3.83 | 0.12 | 3.38 | 0.79, 14.4 | 0.10 |
| Male gender | 0.86 | 0.58, 1.29 | 0.4 | 0.85 | 0.56, 1.29 | 0.4 | 1.61 | 0.71, 3.68 | 0.3 |
| Age category |  |  |  |  |  |  |  |  |  |
| < 5 | — | — |  | — | — |  | — | — |  |
| 5-15 | 2.76 | 1.56, 4.86 | 0.006 | 2.65 | 1.46, 4.81 | 0.001 | 5.84 | 0.75, 45.6 | 0.092 |
| > 15 | 1.39 | 0.85, 2.28 | 0.15 | 1.21 | 0.64, 2.29 | 0.6 | 5.46 | 0.73, 40.6 | 0.10 |
| Improved walls | 0.82 | 0.31, 2.15 | 0.6 | 0.71 | 0.32, 1.57 | 0.4 | 1.01 | 0.29, 3.51 | >0.9 |
| Improved floor | 0.46 | 0.24, 0.91 | 0.032 | 0.51 | 0.30, 0.87 | 0.014 | 0.79 | 0.31, 2.01 | 0.6 |
| Household size | 1.25 | 1.01, 1.56 | 0.043 | 1.19 | 1.08, 1.32 | <0.001 | 0.92 | 0.76, 1.11 | 0.4 |
| Follow-up |  |  |  |  |  |  |  |  |  |
| 0 | — | — |  | — | — |  | — | — |  |
| 1 | 2.35 | 1.18, 4.66 | 0.024 | 2.43 | 1.53, 3.85 | <0.001 | 2.86 | 0.86, 9.50 | 0.086 |
| 2 | 0.93 | 0.31, 2.84 | 0.9 | 0.85 | 0.48, 1.50 | 0.6 | 1.59 | 0.39, 6.54 | 0.5 |
| 3 | 0.74 | 0.38, 1.47 | 0.3 | 0.69 | 0.36, 1.31 | 0.3 | 1.27 | 0.30, 5.41 | 0.7 |
| 4 | 0.55 | 0.18, 1.69 | 0.2 | 0.51 | 0.24, 1.06 | 0.071 | 0.99 | 0.19, 5.02 | >0.9 |
| 5 | 0.16 | 0.04, 0.59 | 0.016 | 0.17 | 0.06, 0.51 | 0.002 | 0.00 | 0.00, 0.00 | <0.001 |
| 6 | 2.13 | 1.09, 4.17 | 0.033 | 1.82 | 0.86, 3.83 | 0.12 | 3.38 | 0.79, 14.4 | 0.10 |
| *^1^*OR = Odds Ratio, CI = Confidence Interval | | | | | | | | | |

| **Supplemental Table 9**. Odds ratios from adjusted models assessing the association between travel variables and incident malaria among cohort members, ensuring travel occurred prior to the malaria diagnosis. | | | | |
| --- | --- | --- | --- | --- |
|  |  | All villages | High transmission villages | Low transmission villages |
|  | Exposure | OR (95% CI) | OR (95% CI) | OR (95% CI) |
| Model 1 | Any overnight travel | 1.49^¥^ (0.88, 2.52) | 1.12 (0.68, 1.85) | 2.09^¥^ (0.79, 5.50) |
| Model 2 | Travel to higher incidence area | 1.84* (1.02, 3.32) | 1.47 (0.84, 2.55) | 2.77^¥^ (0.92, 8.29) |
| Adjusted for sex, age category, household improved floors, household improved walls, household size, and follow-up. ^¥^ < 0.1, *<0.05, ** <0.01, ***<0.001 | | | | |

| **Supplemental Table 10.** Population attributable fractions (percentages) representing the contribution of travel to higher incidence areas on malaria by demographics, overall and by transmission season. | | | | |
| --- | --- | --- | --- | --- |
|  | N | All villages PAF (95% CI) | High transmission villages PAF (95% CI) | Low transmission villages PAF (95% CI) |
| Sex | | | | |
| Male | 821 | 5.8 (1.2, 12.8) | 3.1 (-0.3, 8.5) | 14.9 (4, 33.3) |
| Female | 1097 | 4.9 (1, 11.1) | 2.7 (-0.3, 7.4) | 13 (3.4, 29.8) |
| Age | | | | |
| <5 | 288 | 5.2 (1.1, 11.6) | 2.8 (-0.3, 7.7) | 13.5 (3.6, 30.9) |
| 5-15 | 674 | 2.3 (0.5, 5.5) | 1.2 (-0.1, 3.5) | 6.4 (1.6, 16.4) |
| >15 | 956 | 7.5 (1.6, 16.3) | 4.1 (-0.4, 11) | 18.9 (5.2, 39.9) |
| Occupation (among those > 18 years) | | | | |
| Commerce | 49 | 6.7 (1.4, 14.6) | 3.6 (-0.4, 9.8) | 17 (4.6, 36.8) |
| Farmer | 620 | 7 (1.5, 15.3) | 3.8 (-0.4, 10.3) | 17.8 (4.9, 38.1) |
| Student | 43 | 2.8 (0.6, 6.4) | 1.5 (-0.1, 4.2) | 7.5 (1.9, 18.8) |
| Childcare/homemaker | 13 | 6.7 (1.4, 14.7) | 3.6 (-0.4, 9.9) | 17.1 (4.7, 37) |
| Other | 85 | 13.3 (3, 26.9) | 7.5 (-0.8, 19) | 30.6 (9.4, 55.7) |
| Education level | | | | |
| None | 468 | 5.5 (1.2, 12.2) | 3 (-0.3, 8.1) | 14.2 (3.8, 32.1) |
| Primary | 1052 | 4.3 (0.9, 9.8) | 2.3 (-0.2, 6.4) | 11.4 (3, 26.9) |
| Secondary+ | 394 | 8.1 (1.8, 17.4) | 4.4 (-0.4, 11.8) | 20.1 (5.6, 41.7) |


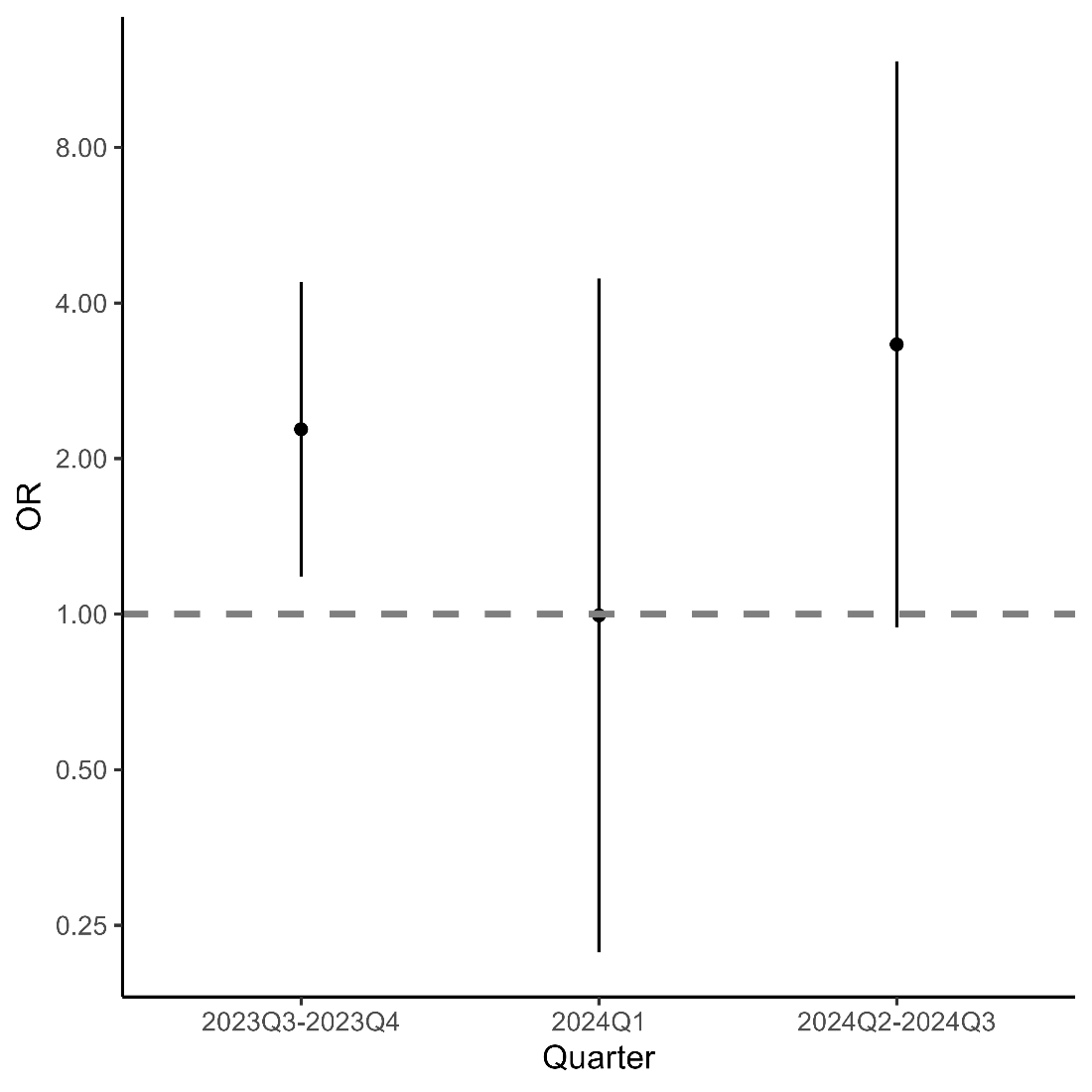


**Supplemental Figure 4.** Relationship between travel to areas of higher incidence (ORs and 95% CI) over time, with only confirmed symptomatic malaria cases and asymptomatic infections as outcome.


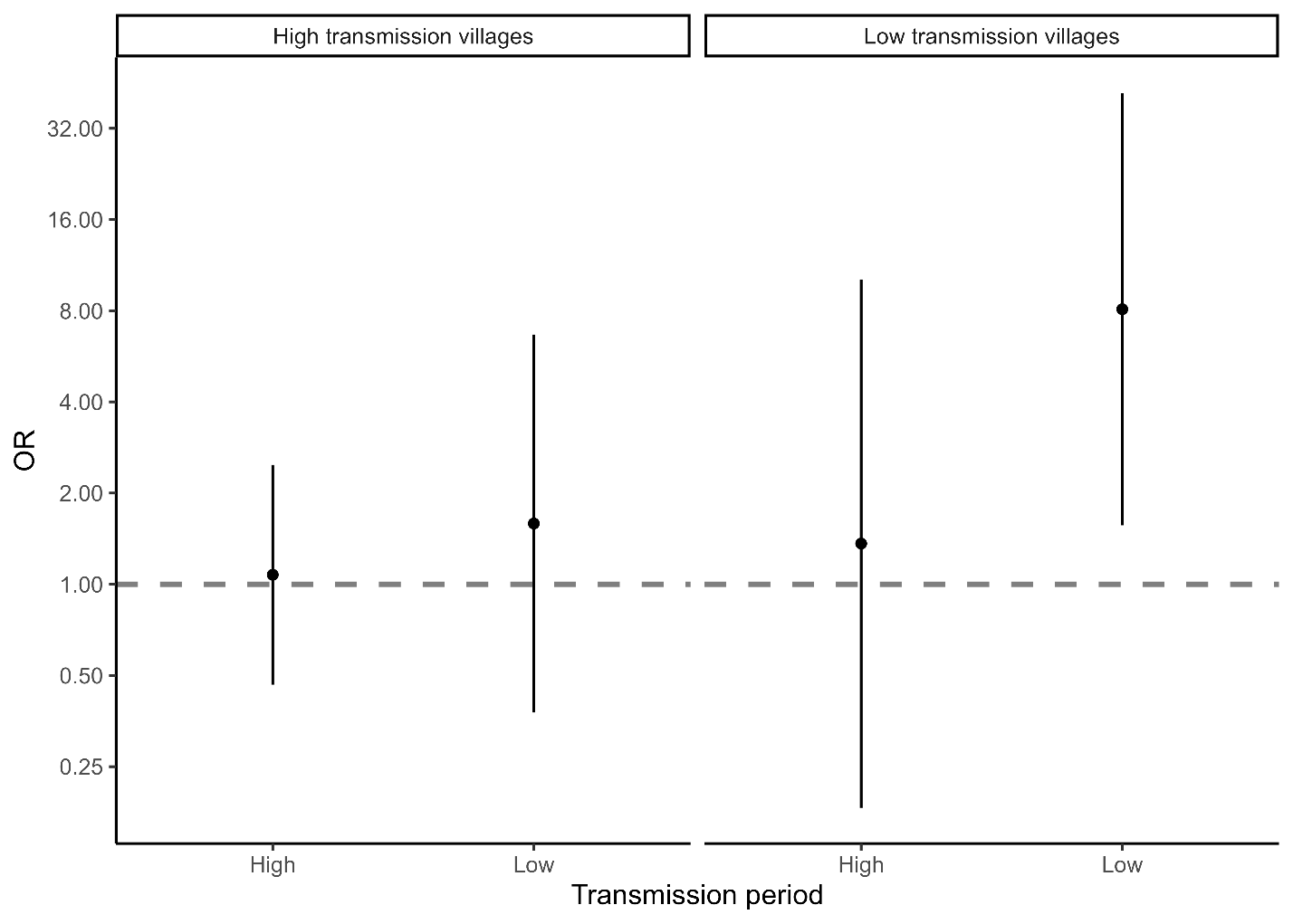


**Supplemental Figure 5.** Relationship between travel to areas of higher incidence (ORs and 95% CI) by time and village-level transmission intensity, with only confirmed symptomatic malaria cases and asymptomatic infections as outcome.
